# Fear of childbirth among pregnant women attending antenatal care in Arba Minch town, southern Ethiopia: a cross-sectional study

**DOI:** 10.1186/s12884-020-03367-z

**Published:** 2020-11-07

**Authors:** Tiruset Gelaw, Teklemariam Gultie Ketema, Kassaw Beyene, Mekdes Kondale Gurara, Gebresilasea Gendisha Ukke

**Affiliations:** 1 Department of Midwifery College of Medicine and Health Sciences, Wollo University, Dessie, Ethiopia; 2 Department of Midwifery College of Medicine and Health Sciences, Arba Minch University, P.O. Box 21, Arba Minch, Ethiopia; 3 School of Public Health College of Medicine and Health Sciences, Arba Minch University, Arba Minch, Ethiopia

**Keywords:** Anxiety, childbirth, Ethiopia, depression, pregnancy, Wijma Delivery Expectancy Questionnaire, stress

## Abstract

**Background:**

Fear of childbirth is one of the life challenges the women encounter during pregnancy. It is an important source of distress for the women and their families and also increases the odds of obstetric complications during childbirth. The aim of this study was to assess the magnitude of fear of childbirth and associated factors among pregnant women attending antenatal care at public health facilities in Arba Minch town, southern Ethiopia.

**Methods:**

Institution-based cross-sectional study was carried out among pregnant women who attended antenatal care at public health facilities in Arba Minch from November 1st − 30th 2019. A systematic random sampling technique was employed to include the participants. Data were collected through a face-to-face interview by using a structured and pretested questionnaire. Wijma Delivery Expectancy Questionnaire was used to score fear of childbirth. Epi Data version 3.1 and Statistical Package for the Social Sciences version 25.0 software were used for data management. Descriptive and analytic analyses were done and statistical significance was declared at a *p*-value < 0.05 and 95% confidence level in multivariable analysis.

**Results:**

A total of 387 pregnant women have participated in this study. Forty (10.3%) of the pregnant women had a low degree fear, 154(39.8%) had a moderate degree fear, 98(25.3%) had a high degree fear, and 95(24.5%) had severe degree fear of childbirth. Unplanned pregnancy (AOR = 2.30, 95% CI: 1.12, 4.74), current pregnancy-related complications (AOR = 6.24, 95% CI: 2.72, 14.29), and poor social support (AOR = 1.93, 95%CI: 1.01, 3.68) were factors significantly associated with severe degree fear of childbirth.

**Conclusions:**

Almost three-fourth of the pregnant women in this study area had moderate to severe degree fear of childbirth. Tailoring counseling during antenatal care visits is needed to address those women who are at a high risk of considerable childbirth fear and its health consequences.

## Background

Childbirth is a normal physiological process that transforms the realities of the mother as well as relationships within and between families and generations[1, 2]. The path of childbirth is associated with both pain and happiness for the women and their families. Women face many challenges from the conception of their babies to the culmination of the childbirth process. Fear of childbirth (FOC) is one of the problems the women encounter during pregnancy and can be a source of distress for the women, their family, and their caregivers [3–5].

Fear of childbirth is described as feelings of uncertainty and anxiousness before, during, or after delivery by thinking the future labor and delivery or experiences of others’ fearful responses to childbirth and labor pain [6, 7]. It is one of the common problems affecting women’s health and wellbeing in their perinatal life [6]. The degree of severity of the fear of childbirth varies from individual to individual ranging from negligible to extreme fear [7–9]. It is estimated that FOC affects about 14% of pregnant women globally and reports from high-income countries indicate that about 10% of pregnant women are suffering from FOC which is of a severe degree[10]. Studies in Africa also showed a high prevalence of FOC ranging from 20–61.2% [11–13].

As one study reported, women with severe FOC seeks more antenatal visit and sick leaves than women with low FOC and the costs for handling women with severe FOC can be as high as 38% than those for women with low FOC [14]. Fear of childbirth has been related to a lot of negative impacts on the physical and psychological wellbeing of a woman: complications during pregnancy, experiencing severe pain during labor and delivery, increased duration of labor, use of anesthesia during labor and increased risk of cesarean deliveries [15–19]. It is also associated with the fear of pain in general and previous difficult labor in particular. Studies indicated that inadequate pain relief during the previous childbirth is the most common reason for requesting a cesarean birth among parous women which can make the women to fear the childbirth process [11, 20]. Women with severe FOC are also vulnerable to subsequent psychological complications such as anxiety and depression [19].

Even though the Ethiopian government focuses on maternal and child health there is still high maternal and infant mortality in the country. As the health care system for women typically focuses on physical health, less attention has been given for the psychological dimensions [21]. Moreover, the available studies about fear of childbirth were conducted in high-income countries and little is known regarding the problem in low-income countries. Since there are differences in cultural values about childbirth from society to society, culturally tailored interventions regarding FOC are required. Thought the problem needs urgent attention, as far as our knowledge is concerned, no study has been conducted in Ethiopia regarding FOC. This study was therefore assessed the magnitude of FOC and factors associated with it among pregnant women attending antenatal care at public health facilities in Arba Minch town.

## Methods

### Study settings, design, period and population

Institution-based cross-sectional study design was employed from November 1st – 30th 2019. The study was conducted in public health facilities in Arba Minch town. Arba Minch is the capital city of Gamo zone in south Ethiopia. The town is located about 495 kilometers south of Addis Abba, the capital of Ethiopia. There were 3 governmental health institutions (1 hospital, 2 health centers), in the town that provide antenatal care(ANC) service for the women in and around the town [22]. This study was conducted at the public health institutions in the town. The source population was all pregnant women who attend antenatal care in public health facilities in Arba Minch town and pregnant women who attended antenatal care service in public health facilities in Arba Minch town during the data collection period were the study population.

### Inclusion and exclusion criteria

Pregnant women who attended ANC service at public health facilities in Arba Minch town were eligible for the study. Women with medical problems or those with pregnancy related complications that affect the women’s ability to give consent and respond to the questions were excluded.

### Sample size determination and sampling technique

The sample size for the first specific objective was determined using single population proportion formula by using the prevalence of FOC from a relevant previous study which was 61.2% (*p* = 0.612) [13] and tolerating a marginal error of 5%, power of 80% (Z_β_= 0.80)and 95% confidence level of certainty (zα/_2_=1.96). As the calculated sample sizes for the second objective (associated factors) were less than that was computed for the first objective, we have used the sample size calculated for the first objective. By using the formula for a single population proportion the calculated sample size was 364.8. By adding 10% for possible non-response rate, the final sample size was 401.28 ≈ **401.**

There are three public health institutions in Arba Minch town, two health centers and one general hospital. According to the information we got from the antenatal care service clinics of the health institutions, about 31 pregnant women visit the hospital and 8 pregnant women visit each of the health centers for antenatal care service daily. The total number of pregnant women who were expected to visit the antenatal care clinics during the data collection period was calculated and used to determine the interval. The total sample size was allocated proportionally to the three public health facilities according to their daily visit load. We then applied a systematic random sampling technique to include every two pregnant women available at the ANC clinics during the data collection period.

### Data collection

Data were collected using structured and pretested questionnaire including Wijma Delivery Expectation/Experience Questionnaire (W-DEQ) and the Oslo social support scale through a face-to-face interview technique. Six midwives were participated in the data collection and supervision after taking training for two days.

The W-DEQ has been designed especially to measure FOC operationalized by the cognitive appraisal of the delivery. This 33-item rating scale has a 6-point Likert scale as a response format, ranging from ' not at all’ (= 0) to ' extremely’ (= 5), yielding a score-range between 0 and 165. Item numbers (2, 3, 6, 7, 8, 11, 12, 15, 19, 20, 24, 25 27, 31) have the reversed score for the calculation of the woman’s individual sum score. Internal consistency and split-half reliability of the W-DEQ was checked and the Cronbach’s alpha score was 0.932.

Degree of childbirth fear was operationalized as: W-DEQ sum score < 38(low degree fear) W-DEQ of 38–65.9 (moderate degree fear), W-DEQ of 66 -84.9 (high degree fear) and W-DEQ ≥ 85 (severe degree fear of childbirth) [23].

Oslo social support scale: is a three-item question which is used to assess psychosocial distress. The sum ranges from 3 to 14 and interpreted as a score of 3–8 (poor social support), 9–11 (moderate social support) and 12–14 (strong social support) [24].

### Data quality assurance and analysis

The questionnaire was first prepared in the English language and translated into Amharic and then translated back to English in order to ensure its consistency. A pretest was done on 20 ANC attendants at another hospital located near the study area. Every questionnaire was checked daily for completeness and accuracy by trained supervisors and principal investigators.

Data were entered into Epi Data version 3.1 after checking for completeness and exported to statistical package for social science (SPSS) version 25.0 for analysis. Descriptive statistical analysis was computed and presented by tables. Binary Logistic regression was used to find out the association between the explanatory and outcome variable. Multivariable Logistic regressions were used to find out the association between dependent and independent variables. Variables with a p-value of less than 0.25 in binary logistic regression were considered for the multivariate logistic regression analysis. A Multicollinearity test was done to check the correlation between independent variables. The model goodness of fit was tested using Hosmer and Lemeshow test which was 0.910. The statistical association was evaluated using odds ratio at 95% confidence interval and a p-value less than 0.05.

## Results

### Socio demographic status of the study participants

A total of 387 pregnant women were interviewed yielding a 96.5% response rate. The age of the participants was ranged from 18 to 42 years with the mean age of 27 ± 5 years. (Table 1).

**Table 1:** Socio-demographic status of pregnant women attended ANC at public health facilities in Arba Minch town, Southern Ethiopia, 2019 (*n* = 387)

Variable	Category	Frequency	Percent
Age in years	18–19	14	3.6
	20–24	121	31.3
	25–29	127	32.8
	30–34	97	25.1
	35–39	21	5.4
	40–45	7	1.8
Residence	Urban	288	74.4
	Rural	99	25.6
Marital status	Single	21	5.4
	Married	364	94.1
	Divorced	2	0.5
Occupational status	Employed	124	32.0
	House wife	180	46.5
	Self-employed	54	14.0
	Daily laborer	9	2.3
	Student	12	3.1
	Other^a^	8	2.1
Educational status	No formal education	28	7.2
	Primary education	117	30.2
	Secondary education	106	27.4
	College and above	136	35.2
Religion	Orthodox Christian	175	45.2
	Protestant Christian	168	43.4
	Muslim	24	6.2
	Catholic	17	4.4
	Others^b^	3	0.8
Ethnicity	Gamo	190	49.1
	Gofa	91	23.5
	Wolayta	45	11.6
	Amhara	25	6.5
	Oromo	22	5.7
	Other^c^	14	3.6

^a^wood collecting, farmer, jobless, other ^b^Adventist, Jehovah^,^s witness, ^c^Konso, Gurage, Tigrie, Sidama, Zeyse

### History of abuse and social support status

Twenty-three (5.9%) of the participants reported that they had a history of abuse in their lifetime and 191(49.3%) had a husband’s support during the current pregnancy. With regards to social support: 102(26.3%) had poor social support, 191(49.3%) had moderate social support and the rest 94(24.3%) had high social support.

### Obstetrics characteristics of the participants

More than half (52.5%) of the study participants were in the third trimester of their pregnancy. The majority (87.2%) of the parous women had given birth to the preceding baby via spontaneous vaginal delivery (Table 2).

**Table 2:** Obstetrical characteristics of the study participants, Arba Minch town, Southern Ethiopia, 2019 (*n* = 387)

Variable	Response	Frequency	Percentage
Gravidity	Primigraviada	122	31.5
	Multigravida	265	68.5
Parity	Nulliparous	129	33.3
	Primiparous	142	36.7
	Multiparous	116	30
Trimester	First trimester	03	0.8
	Second trimester	181	46.7
	Third trimester	203	52.5
Mode of delivery of the preceding pregnancy (n = 258)	Vaginal birth with episiotomy	74	28.7
	Vaginal birth without episiotomy	151	58.5
	Instrument assisted vaginal birth	12	4.7
	Caesarean delivery	21	8.1
Planned current pregnancy	Yes	348	89.9
	No	39	10.1
Wanted current pregnancy	Yes	373	96.4
	No	14	3.6
Problems during the current pregnancy	Yes	28	7.2
	No	359	92.8
Had history of abortion	Yes	17	4.4
	No	370	95.6
Obstetrical/medical problems during the previous pregnancy(n = 265)	Yes	29	10.9
	No	236	89.1
Had history of stillbirth	Yes	13	3.4
	No	374	96.6
Had fear of medical intervention	Yes	270	69.8
	No	117	30.2
Husband support present	Yes	355	91.7
	No	32	8.3

### Magnitude of fear of childbirth

Forty (24.3%) of the pregnant women had W-DEQ sum score < 38(low degree fear), 154(39.8%) had W-DEQ score of 38–65.9 (moderate degree fear), 98(25.3%) had a W-DEQ score of 66–84 (high degree fear), and 95(24.5%) had severe degree fear of childbirth (W-DEQ ≥ 85).

### Factors associated with severe degree fear of childbirth

As the health consequences of sever degree childbirth fear likely to be of significant importance, we have executed bivariate and multivariable analysis for severe degree fear of childbirth. Accordingly, the bivariate analysis showed educational status, parity, unplanned pregnancy, current pregnancy complications, no husband support for pregnancy and poor social support were factors associated with sever degree fear of childbirth. In multivariable logistic regression analysis, unplanned pregnancy, current pregnancy problems and poor social support were factors associated with severe degree fear of childbirth. Those women who had unplanned pregnancy were about 2 times more likely to have severe degree fear of childbirth compared with those with planned pregnancy (AOR = 2.30, 95% CI: 1.12, 4.74). Women who had current pregnancy-related complications were about 6 times more likely to have severe degree fear of childbirth than those without current pregnancy-related problems (AOR = 6.24, 95% CI: 2.72, 14.29). Moreover, participants who had poor social support were about 2 times more likely to report severe degree fear of childbirth (AOR = 1.93, 95%CI: 1.01, 3.68) (Table 3).

**Table 3:** Multivariable logistic analyses for severe degree fear of childbirth and associated factors among pregnant women, Arba Minch town, southern Ethiopia, 2019

	Variables	Severe degree Fear of childbirth		COR 95%CI	AOR 95%CI
		Yes (n, %)	No (n, %)		
Educational status	No formal education	10(35.7%)	18(64.3%)	1.88(0.79, 4.5)	0.96(0.36, 2.55)
	Primary education	31(26.5%)	86(73.5%)	1.22 (0.69, 2.17)	0.60(0.22, 1.61)
	Secondary education	23(21.7%	83(78.3%)	0.94(0.509, 1.73)	0.79(0.30, 2.09)
	College and above	31(22.8%)	105(77.2%)	1	1
Parity	Nulliparous	38(29.5%)	91(70.5%)	1.47(0.91, 2.38)	1.61(0.94, 2.74
	Multiparous	57(22.5%)	201(77.9%)	1	1
Planned pregnancy	No	16(41%)	23(59%)	2.34(1.2, 4.70*	2.30(1.12, 4.74)*
	Yes	79(22.7%)	269(77.3%)	**1**	**1**
Current pregnancy complication	Yes	17(60.7%)	11(39.3%)	5.57(2.5, 12.34)**	6.24(2.72, 14.29)**
	No	78(21.75%)	281(78.3%)	1	1
Husband support	No	11(34.4%)	21(65.6%)	1.7(0.78, 3.65)	0.84(0.36, 1.99)
	Yes	84(23.7%)	271(76.3%)	1	1
Social support	Poor support	38(37.3%)	64(62.7%)	1.94(1.04, 3.63)*	1.93(1.01, 3.68)*
	Moderate support	35(18.3%)	156(81.7%)	0.73(0.40, 1.34)	0.66 (0.35, 1.24)
	Strong support	22(23.4%)	72(76.6%)	1	1

*p-value<0.05, **p-value=0.001, *AOR *Adjusted Odd Ratio, *COR *Crude Odd Ratio, *CI *Confidence interval

## Discussion

In this study fear of childbirth among pregnant women attending ANC services in public health facilities in Arba Minch town was investigated. Since almost all pregnant women had some degree of childbirth fear, we used a W-DEQ sum score of 85 or more as women having a severe degree (considerable) fear of childbirth and the discussion was based on this cut-off point. Accordingly, the proportion of women with a considerable fear of childbirth in this study was 24.5% (95%CI: 20%, 29%). The finding from this study is similar to other studies conducted in Malawi (20%), Iran (20%) and Australia (24%) [5, 12, 25]. However, the cut-off points used in this study (≥ 85) and studies conducted in Malawi (≥ 66) and Australia (≥ 66) is different indicating that the burden of the problem is still higher among women in this study area. It is also higher than the findings from a study which included six European countries and has used similar cut-off point for severe degree of childbirth fear where the prevalence of FOC ranged from 4.5 among primigraviadae in Belgium to 15.6 among primigraviadae in Estonia[26] and a result from a recent study conducted in Kenya where the prevalence of severe degree of childbirth fear was 8% [27]. On the contrary, findings from studies conducted in India (45.4%),[28] the United States of America (39.4%)[29] and Turkey (82.6%)[25] have shown higher burden of the problem than results from this study.

These discrepancies may be due to the differences in culture and attitudes towards childbirth, differences in health institutions structure, quality of ANC, and the difference in tools used to measure the degrees of fear. Moreover, the inconsistency with the cut-off points to define the degree of childbirth fear can also affect the difference in the magnitude of the problem. For instance, some studies used 50, other 66, some 85 as cut-off point [26–28]. Overall we can see that fear of childbirth is common among pregnant women across the world but the way the problem is reported has subjectivity which needs common consensus.

In the current study, unplanned pregnancy was strongly associated with FOC, which is supported by findings from a study conducted in Turkey, a study from six European countries and a study from Bangkok[26, 30–32]. The reason behind this may be due to an increase in stress among women with an unplanned pregnancy in addition to pregnancy physiological maladaptation. It is likely that the pregnancies are close to the previous ones that there might be obstetrical cases that demanded spacing the regencies. Unplanned pregnancy means the woman has more likely to have another life plan and the co-existence of the two conditions might have increased the degree of fear among these women. This is also supported by another study conducted in Turkey where women with unplanned pregnancy had problems with adaptation to pregnancy, felt more pain during labor and were at high risk of depression during puerperium [33, 34].

The other factor which had significant association with FOC in this study was the presence of complications during pregnancy. Women who had pregnancy-related complications during the current pregnancy have more likely to have a fear of childbirth than those who were not. This finding is supported by study findings from a study conducted in Turkey [31]. The possible justification may be due to inadequate individualized counseling that women should get and stress from rumors and information they gather from the previous experience or other women or media and thus increases level fear of childbirth. Fear of being dying, baby injury, poor control of the body, lack of trust in health care professionals, lack of confidence to give birth and others may also increase if adequate counseling was not given to those women with pregnancy-related complications. Other study also reported the positive correlations between childbirth fear, and other problems during pregnancy [35].

Moreover, pregnant women who had poor social support were more likely to have FOC. This is in line with the study done in six European countries (Belgium, Iceland, Denmark, Estonia, Norway, and Sweden), Finland and the United States [26, 29, 36]. Another study conducted in Norway also supports the findings [37]. The possible justification may be due to the strong support by family, neighbor, or midwives can strengthen women’s belief that childbirth is a physiological and controllable process, and thus, result in psychological wellbeing and reduced fear of childbirth. For women who had support from husband, family, mother or mother-in-law together with a trained midwife may reduce fear.

Bringing fear of childbirth into the light as a problem among Ethiopian women may be taken as the strength of the study as most of the people including the women in the country themselves have not been considering the situation as a problem. However, the main limitation of this study was that we could not assess some variables which might have an important association with the problem like the history of abuse/violence during pregnancy with the tool.

Overall, the findings from this study conveys a clear message to health managers and health persons working in the maternity area about where, when and how to act in order to reduce morbidities related to childbirth fear.

## Conclusions

This finding showed a high magnitude of fear of childbirth in pregnant women in this study area. Complications in the current pregnancy, poor social support and unplanned pregnancy were the factors significantly associated with fear of childbirth. There should be special attention and counseling for pregnant women who have an unplanned pregnancy and pregnancy-related complications such as mal-presentations, pregnancy-induced hypertension, gestational diabetes mellitus, antepartum hemorrhage and multiple gestation. Early identification of expectant mothers with severe childbirth fear during pregnancy by health-care professionals to provide cognitive behavioral therapy and support for their psychological and physical health is important to decrease the next FOC complications. We strongly suggest family planning providers as well as ANC providers to give appropriate information about how to avoid unplanned pregnancies and what to do when this happened. As Ethiopia is a multi-ethnic country, we recommend more studies that include mixed methods and cover a wider area. We also suggest for researchers across the world to use consistent tools to evaluate fear of childbirth to have comparable findings and also conducting a systemic review in the area.

## Data Availability

The datasets used and/or analysed during the current study available from the corresponding author on reasonable request.

## Abbreviations

ANC: Antenatal care
FOC: Fear of Childbirth
IQR: Interquartile range
SD: Standard deviation
W-DEQ: Wijma Delivery Expectation/Experience Questionnaire
